# The *BIP* software: high-level abstraction for reproducible biological image analysis

**DOI:** 10.1093/bioinformatics/btag665

**Published:** 2026-09-24

**Authors:** Eric Biot, Sandrine Lefranc, Ayoub Ouddah, Erwan Guerrier, Jasmine Burguet, Philippe Andrey

**Affiliations:** Université Paris-Saclay, INRAE, AgroParisTech, Institute Jean-Pierre Bourgin for Plant Sciences, 78000, Versailles, France; Université Paris-Saclay, INRAE, AgroParisTech, Institute Jean-Pierre Bourgin for Plant Sciences, 78000, Versailles, France; Université Paris-Saclay, INRAE, AgroParisTech, Institute Jean-Pierre Bourgin for Plant Sciences, 78000, Versailles, France; Université Paris-Saclay, INRAE, AgroParisTech, Institute Jean-Pierre Bourgin for Plant Sciences, 78000, Versailles, France; Université Paris-Saclay, INRAE, AgroParisTech, Institute Jean-Pierre Bourgin for Plant Sciences, 78000, Versailles, France; Université Paris-Saclay, INRAE, AgroParisTech, Institute Jean-Pierre Bourgin for Plant Sciences, 78000, Versailles, France

## Abstract

**Motivation:**

The bioimage informatics community is increasingly facing needs for the reproducible processing and analysis of large batches of multi-dimensional images through complex pipelines. We introduce BIP, a new open-source software for addressing these needs. The distinctive features of BIP is to be natively designed for batch and pipeline processing, relying on a unified, simple, and human-readable high-level syntax for processing single or multiple images and for specifying individual or combined operations. As a command-line tool, BIP also lends itself seamlessly to high-performance computing applications. BIP invests an original niche in the ecosystem of bioimage informatics software and should contribute to the development of reproducible research in bioimage processing and analysis.

**Availability:**

BIP is distributed as an open source software under the GNU General Public License, version 3. The source code is available at https://forge.inrae.fr/andreylab/bip and can also be found at the Software Heritage permalink https://archive.softwareheritage.org/swh:1:dir:dded82e71041943bdfdf7b4684c69dd742c67312. Executables for Linux and Windows are available from the BIP website at https://andreylab.versailles.inrae.fr/html/bip.html. The distribution also includes a detailed user manual (PDF and HTML formats). To enable users to identify the right operators for their tasks, the principles and effects of the operators are briefly exposed, and many illustrations are provided. The manual also includes a tutorial section, illustrating how to solve typical, commonly encountered problems in bioimage analysis. The material for the tutorials is publicly available at https://doi.org/10.57745/JIWVCE.

## 1. Introduction

Extracting quantitative information from microscopy images requires the design and application of image processing pipelines composed of elementary operations such as background subtraction, noise reduction, object segmentation and morphological filtering. Current trends towards reproducible research have underscored the need for reproducibility and replicability in bioimage analysis (Aaron and Chew 2021, Miura and Nørrelykke 2021). In addition to the requirement for portable software, with containerisation as one solution, this has two major implications. First, users must be able to reproduce published results on a given dataset (data reproducibility). Second, the same pipeline should be applicable to new datasets to generate new results (pipeline reproducibility). Achieving both goals requires a clear and complete separation, in image analysis workflows, between the data and the operations applied to these data. With few exceptions, this requirement has seldom received attention and is even less satisfied by existing bioimage analysis tools (Gilles and Boudier 2020).

The ultimate corollary of separating pipelines from data is the need to specify both datasets and pipelines in a generic manner. However, the structure of biological image datasets is complex and hierarchical. They typically contain tens to hundreds or thousands of individuals images. Images are themselves single 2D or 3D frames, or contain multiple channels (color or fluorescence channels) and/or multiple timepoints (time-lapse imaging). Pixels may be scalar or vector-valued, as when computing Fourier transforms or gradients, and they can be encoded using diverse numerical types, from 8-bit integers to double precision floats. Likewise, processing an image can involve a single operation or a complex sequence of operations with their parameters.

Existing bioimage analysis tools fall within two categories. On the one hand, dedicated libraries such as ITK (Yoo *et al.* 2002), DIPLib (https://github.com/DIPlib/diplib) or ImgLib2 (Pietzsch *et al.* 2012) provide collections of basic components that can be assembled into dedicated, possibly complex pipelines. This requires a strong expertise in low-level computer programming, such as C++. In addition, the programmer is exposed to the complexity of handling various image types (2D, 3D, etc.) and numerical formats (8-bit, 16-bit, etc.). On the other hand, integrated software such as ImageJ/Fiji (Schindelin *et al.* 2012, Schneider *et al.* 2012), Icy (de Chaumont *et al.* 2012), or CellProfiler (Stirling *et al.* 2021) provide user-friendly graphical interfaces that allow end-users to perform a large spectrum of predefined image operations. Carefully designed tools also allow users graphically define and run pipelines in this ecosystem (Dietz and Berthold 2016, Stirling *et al.* 2021, Gerst *et al.* 2023, Cross *et al.* 2024). However, the support for different image structures and numerical formats as well as the possibilities for automation and batch processing can be limited. For example, not all operators and plugins in the ImageJ/Fiji ecosystem support all image types and numerical formats and it is only recently that some support to 3D images was introduced in CellProfiler (McQuin *et al.* 2018). Manipulating complex image structures requires writing specific code, for example loops to iterate over frames in time-lapse images. Last, these tools may not be compatible with high-performance computing on remote or cloud servers (Paul-Gilloteaux *et al.* 2021). Altogether, exposing users to the complexity of image structure and pipeline specification requires programming skills and a significant time of development, while representing a potential source of errors. This is a major obstacle to efficiency, robustness, and reproducibility.

Ideally, users should concentrate on the logic of their analyses (*what* has to be done), not on how to achieve them (*how* to do it). Similarly, they should focus on *which* data to process, not on *how* to specify their batch contents. This requires that both datasets and pipelines be specified at a high level of abstraction.

Here, we introduce such an abstraction and its implementation in a new software, BIP (Biological Image Processing), a general purpose image processing and analysis tool combining versatility, efficiency, and easy batch processing capability. The two categories of tools described above represent two ends along the continuum of computational solutions to image processing problems (Haase *et al.* 2022). BIP invests an intermediate niche, retaining the performance and versatility of low-level tools and the user-friendly simplicity of integrated software. We review below the main features of BIP, illustrate its potential for reproducible, large scale image processing and analysis, and compare its performances to state-of-the-art alternatives.

## 2. Results

BIP was designed as a command-line tool, taking inspiration from Unix-like systems and benefiting from shell functionalities to specify dynamically (i) complete pipelines resulting from the combinations of elementary operators and (ii) the sets of image files to process. Hence, the major distinctive feature of BIP is to be natively batch and pipeline oriented, abstracting single image and batch processing under a unifying concept, and similarly, abolishing the distinction between atomic operations and pipelines. There are currently 180+ operators in BIP, covering the whole range of image processing and quantitative analysis tasks (point operations, spatial filters, complex transforms, segmentation, mathematical morphology, morphological and intensity measurements, topological analyses, etc.), including operators to invoke deep learning tools such as PlantSeg/PanSeg (Wolny *et al.* 2020) or CellPose-SAM (Stringer *et al.* 2021, Pachitariu *et al.* 2025).

**Figure 1:**
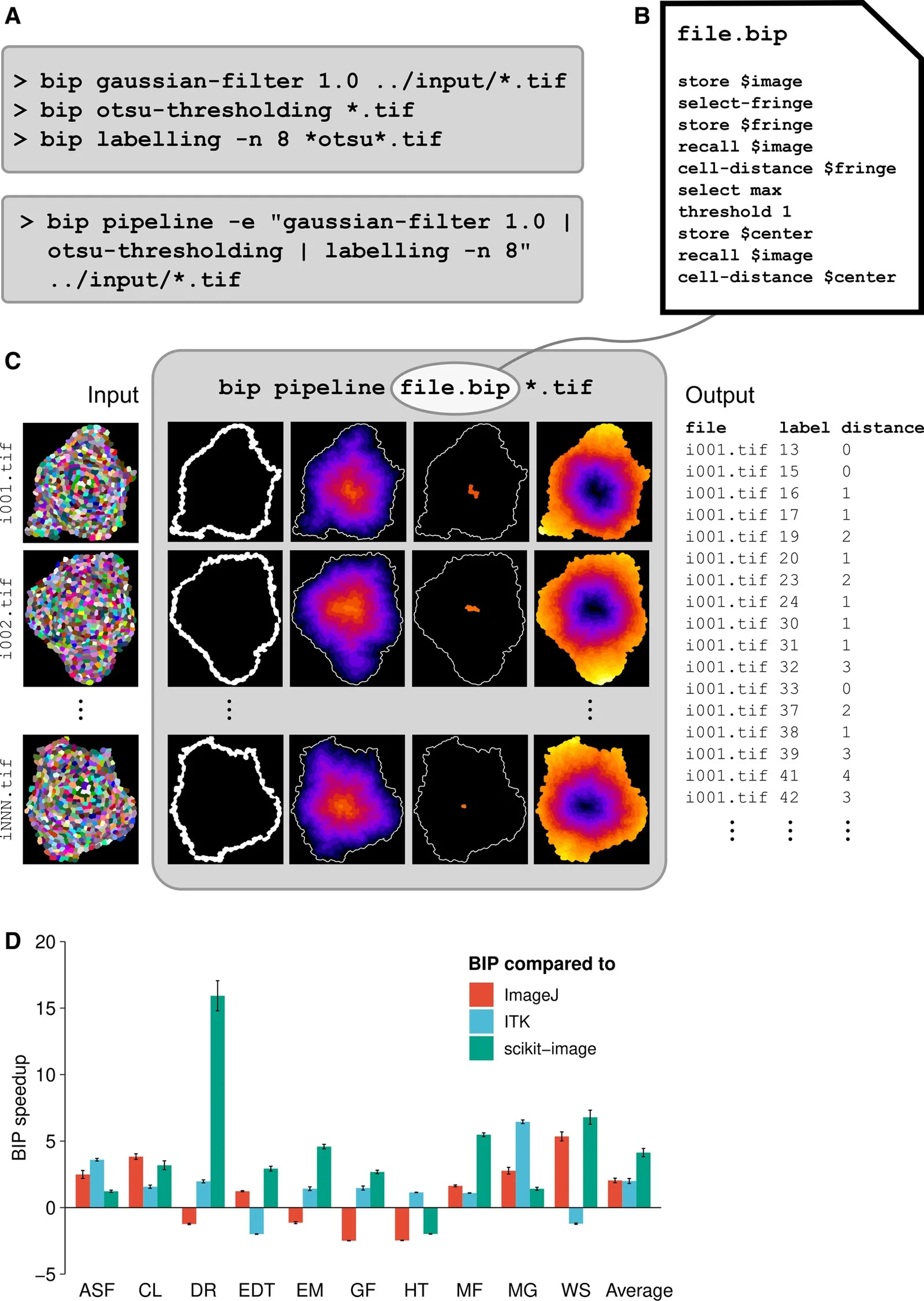
Batch processing with BIP. (A) Operators can be individually invoked from the command-line and applied to one or several images selected using shell wildcards (*Top*). They can also be assembled on-the-fly into pipelines (*Bottom*). (B) Pipelines can be scripted into text files. The example pipeline computes the cell-to-center distance for all segmented cells within a tissue. (C) Application of the pipeline shown in (B) to a batch of input images containing segmented cells in different plant shoot apical meristems. The thumbnail images illustrate some steps along the pipeline (2D projections of 3D images). The final image is a map of cell-to-center distance. Color-coding was performed in Fiji (Schindelin *et al*. 2012). (D) BIP speedup with regards to other image processing tools. ASF: alternate sequential filter; CL: component labelling; DR: dilation reconstruction; EDT: Euclidean distance transform; EM: extended minima; GF: Gaussian filter; HT: hysteresis thresholding; MF: median filter; MG: morphological gradient; WS: watershed transform. Bars: mean ± s.e.m. (n = 5).

BIP can be used to apply one or more operations to one or more images. Applying a single operation is achieved by simply calling BIP with the corresponding operator name and parameters, if any, followed by one or several image filenames (Fig. 1A, *Upper box*). Unifying the syntax for processing single or multiple images makes BIP a naturally batch-oriented software. Passing filenames as arguments on the command line allows using shell wildcards to select the images to process. All BIP executions are automatically logged, providing a trace of operators, parameters, and processed images, which is essential for reproducibility.

Image analysis frequently requires that different images be associated or combined. For example, measuring average intensities in objects requires pairing raw images with their segmented counterparts; likewise, selecting objects contained in other ones requires masking contents between segmented images. BIP can automatically associate images with matching filenames across directories (Fig. S1). Substitution patterns mapping input filenames to paired filenames can be specified using regular expressions (Fig. S2). Hence, an arbitrary number of image pairs can be processed in a single call, which facilitates automation and contributes to robustness when achieving complex tasks. An additional benefit is to enforce the adoption of standardized file naming conventions and the rigorous organization of image data on disk, as has been proposed with the BIDS initiative in the neuroimaging community (Gorgolewski *et al.* 2016). Such standards, which are key requirements for the application of FAIR principles and for reproducible image analysis, are likely to spread within the microscopy community (Bourget *et al.* 2022).

A key distinctive feature of BIP is how easily pipelines of sequential operations can be defined and applied to sets of images. A pipeline is simply defined by listing its elementary operations in a text file, which is passed to the *pipeline* operator. Pipeline files follow the same simple, human language-like syntax as command line calls. This makes pipeline writing straightforward and reduces the learning cost. One benefit of using pipeline files is to ease the design of complex workflows and to provide a natural mechanism for tracing operations. Another advantage of executing pipelines rather than separate operations one by one is a reduced processing time. Because intermediate images are kept in memory, the costly read/write disk operations that can dominate runtime are avoided. If needed, an option can force the saving of intermediate steps during pipeline execution, which is particularly useful for tracing and debugging. Lastly, the lightweight, data-independent format used to manipulate pipelines facilitates their sharing across laboratories, further promoting the adoption of FAIR principles.

Pipelines can also be specified as expressions on the command line rather than as text files. The same syntax is used as in command line calls of single operators and in pipeline files, using the Unix pipe symbol (“|”) to separate successive operators (Fig. 1A, *Lower box*). This is particularly convenient for simple pipelines that do not require being saved as permanent files. This is also useful when designing new pipelines, where trial and error attempts can be performed repeatedly to setup short sequences of operations or to tune parameters.

Complex image processing pipelines frequently require branching between sequences of operations to be executed in parallel. To support this, pipeline-specific operators allow manipulating variables to store and retrieve images at given steps in a pipeline. Variables can also be used to dynamically load images related to the currently processed images, using the same filename pattern matching mechanism described above for pairing images. This mechanism further contributes to the adoption of good practices for naming files and organizing data.

Thanks to the abstraction of atomic operations and pipelines under the same unifying concept, BIP pipelines can invoke other BIP pipelines. This favors modular design of complex pipelines, enforces self-documentation through the filenames given to the different sub-pipelines, simplifies pipeline debugging and maintenance, and allows reuse of existing pipelines.

An illustration of how complex image analysis tasks can be easily specified in BIP using a compact, human understandable and editable high-level syntax is given in Fig. 1B. This pipeline computes the cell-to-cell distances from all segmented cells in a tissue image to the center of the tissue. Figure 1C shows how a simple shell instruction suffices to invoke this pipeline on an arbitrary number of images.

Thanks to its abstraction of different image structures under a single unifying concept, BIP transparently supports images of any spatial dimension (2D or 3D), with any number of channels and time points. Vector-valued images, such as true-color, gradient or complex-valued images are also supported. Overall, BIP handles a wide range of 2D to 6D multi-dimensional images (Table S1). By default, all channels and timepoints are processed but the processing scope can be modified, for example to apply distinct pipelines to different channels. BIP supports all standard numeric types, from signed and unsigned integers (8, 16, and 32 bit) to floating-point numbers (single and double precision). This allows setting an optimal balance between image file size and the numerical representation required for the desired processing and numerical accuracy. To support this exceptionally large range of numerical types, BIP takes as input images stored in the TIFF format (and derived formats such as OME-TIFF or Zeiss LSM). TIFF is among the most popular image file formats supporting all these data types for all image dimensions (2D, 3D, 2D + t, etc.), and all other bioimage formats can be converted to TIFF using the Bio-Formats tool (Linkert *et al.* 2010).

BIP is written in C++ based on the Free-D software library suite (Biot *et al.* 2016). The image library classes rely on C++ templates to support the processing of images with different numeric types. We implemented a specific mechanism to automatically invoke the appropriate template without knowing a priori the image numeric type. Image values are stored as 1D arrays, with neighbourhoods defined by 1D offsets on these arrays. This allows to write generic code independent of numeric type and dimensionality, thus reducing costs in implementation, optimization, and maintenance. Thanks to these specific design features, the code of any BIP operator transparently handles 2D and 3D images, static and time-lapse images, 8-bit or float images, etc. Altogether, adding a new operator to BIP from an existing image processing or analysis class takes a few minutes only, ensuring evolutivity and ease of maintenance. Many operator implementations are multi-threaded, allowing users to take advantage of the computational power of HPC servers. As a command-line tool, BIP also seamlessly lends itself to HPC usage through schedulers such as SLURM (Yoo *et al.* 2003) or GNU Parallel (Tange 2011). BIP is also compatible with workflow management systems, including GNU Make (Baker 2020), Snakemake (Köster and Rahmann 2012), NextFlow (Di Tommaso *et al.* 2017), and Galaxy (Abueg *et al.* 2024), a key feature for automation and reproducibility (see Tutorials for examples).

We benchmarked BIP performance against popular image processing tools (Fiji, ITK, and scikit-image). We selected 10 operators covering linear and non-linear filtering, thresholding or region-based segmentation, and mathematical morphology (Table S2). These operators were run on images of various dimensions, size, and modalities (Fig. S3 and Table S3). Overall, BIP was the fastest or the second fastest of the four tools. On average, it was about 2.5 times faster than Fiji and ITK and about 5 times faster than scikit-image (Fig. 1D).

## 3. Conclusion

BIP provides a command-line tool with a rich set of operators that can be seamlessly assembled into complex pipelines to process and analyze multi-dimensional images. It fills a niche that has remained largely unexplored in the ecosystem of bioimage informatics software, and only rarely invested in other fields such as image manipulation and computer vision (ImageMagick Studio LLC 2024, https://imagemagick.org) (Tschumperlé *et al.* 2025). BIP is not intended to replace existing tools but rather to complement them. For example, many tasks that can be automated will be easier to perform in batch with BIP; conversely, visualization and highly interactive tasks will be best performed using GUI-based software. As a natively batch-oriented tool, BIP should be of interest to bioimage informaticians as well as biologists confronted with repetitive analysis needs over large image datasets. Relying on command line interface is not yet common practice in bioimage analysis. BIP allows the corresponding learning cost to be minimized. Many biologists have already engaged in using command line tools for bioinformatics analyses (Brandies and Hogg 2021). With the increasing needs to process large image datasets in a reproducible way, we anticipate a similar trend in bioimage informatics. We believe BIP should be a valuable tool to foster and to accompany this transition.

## Supplementary Material

btag665_Supplementary_Data

## Data Availability

No new data were generated or analysed in support of this research.
