# Supplementary material for: The BIP software: high-level abstraction for reproducible biological image analysis
Source: Bioinformatics. 2026 Sep 24;42(9):btag665. doi: 10.1093/bioinformatics/btag665 (PMC13614421; doi:10.1093/bioinformatics/btag665)
Supplement: Supplementary file 1 [file btag665_supplementary_data.pdf]

Supplementary information

The *BIP* software: high-level abstraction for reproducible biological image analysis

Eric Biot, Sandrine Lefranc, Ayoub Ouddah,
Erwan Guerrier, Jasmine Burguet and Philippe Andrey*

Université Paris-Saclay, INRAE, AgroParisTech,
Institute Jean-Pierre Bourgin for Plant Sciences,
78000, Versailles, France

* philippe.andrey@inrae.fr

Benchmarking

We compared BIP execution times with three other software packages: ImageJ/Fiji (Schindelin et al., 2012) equipped with the MorphoLibJ plugin for mathematical morphology (Legland et al., 2016), the Insight Toolkit ITK (Yoo et al., 2002; McCormick et al., 2014), and scikit-image (van der Walt et al., 2014). The benchmark was performed by applying ten image processing operators (Table S2) to image batches acquired using different biological imaging modalities and covering a wide range of spatial scales from the organelle to the organ (3D confocal microscopy, 3D-SIM, Serial block-face electron microscopy). Each modality was represented by three images of different sizes (Figure S3 and Table S3). Each operator was run five times on each image batch. For each software included in the benchmark, we computed the ratio between the execution time of an operator under this tool and the corresponding average execution time under BIP. The BIP speedup was defined as the value of this ratio when it was > 1. Otherwise, the BIP speedup was taken as the negative inverse value of the ratio.

For each software, the processing was invoked from a shell script in a terminal, executing each algorithm and returning the execution time. The versions of the programs used were ImageJ 1.54f, ITK 5.2.1-3, and scikit-Image 0.24.0. The comparison was carried out on a Dell Inc. Precision 3530 based on Intel® Core™ i7-8850H CPU @ 2.60GHz × 12, with 32GB RAM and running Ubuntu 22.04.4LTS. The codes and scripts used in this benchmark are available at `https://forge.inrae.fr/andreylab/bip-benchmarking`.

space	channels	timepoints	values	numeric types
2D	1	1	1	all
2D	K	1	1	all
2D	1	T	1	all
2D	1	1	V	all
2D	K	T	1	all
2D	K	1	V	all
2D	1	T	V	all
2D	K	T	V	all
3D	1	1	1	all
3D	K	1	1	all
3D	1	T	1	all
3D	1	1	V	all
3D	K	T	1	all
3D	K	1	V	all
3D	1	T	V	all
3D	K	T	V	all

Table S1: Image types supported in BIP. All combinations of spatial dimensions, number of channels (1 or $K > 1$), of timepoints (1 or $T > 1$), of values per image element (1 for scalar images or $V > 1$ for vector images) are supported. For each of the 16 combinations, all of the 8 numeric types (signed and unsigned integers in 8, 16 and 32 bits and floating-point numbers in single or double precision) are supported. Overall, 128 image types are supported by BIP.

**Operators**	**Telomeres**	**Roots**	**Meristems**	**Spores**
Alternate sequential filter				
Structuring element shape	Cube	Cube	Cube	Square
Structuring element radius	1–3	1–3	1–3	1–3
Euclidian distance transform	NA	NA	NA	NA
Extended minima				
Dynamics	200	10	5	500
Connectivity	6	6	6	4
Gaussian filter				
Sigma (pixels)	3	3	3	3
Hysteresis thresholding				
Low threshold	300	30	180	14500
High threshold	500	150	200	14900
Connected component labeling				
Connectivity	6	6	6	4
Median filter				
Neighbourhood shape	Cube	Cube	Cube	Square
Neighbourhood radius	3	3	3	3
Morphological gradient				
Structuring element shape	Cube	Cube	Cube	Square
Structuring element radius	1	1	1	1
Reconstruction by dilation				
Connectivity	6	6	6	4
Marked-based watershed				
Connectivity	6	6	6	4

Table S2: Benchmark study: selected operators and their parameterization for the different images. NA indicates the operator has no parameter.

Images	Modality	XYZ sampling (µm)	Depth	XYZ size
HeLa 1.2.11 cells	3D-SIM	0.04, 0.04, 0.125	16 bits	474,538,65
Telomeres				561,505,95
(Keller et al., 2024)				450,446,85
Arabidopsis thaliana	3D Confocal	0.18, 0.18, 0.18	8 bits	1024,1024,261
Shoot apical meristem				1024,1024,228
(SPACE team, IJPB)				1024,1024,270
Arabidopsis thaliana	3D Confocal	0.345, 0.345, 0.35	8 bits	1022,444,388
Root tips				1023,449,388
(SPACE team, IJPB)				869,537,361
Anncaliia algerae	SBF-SEM	0.0022, 0.0022	16 bits	10000,10000
Spores				10000,10000
(Chang et al., 2023)				10000,10000

Table S3: Image collection used for benchmarking.

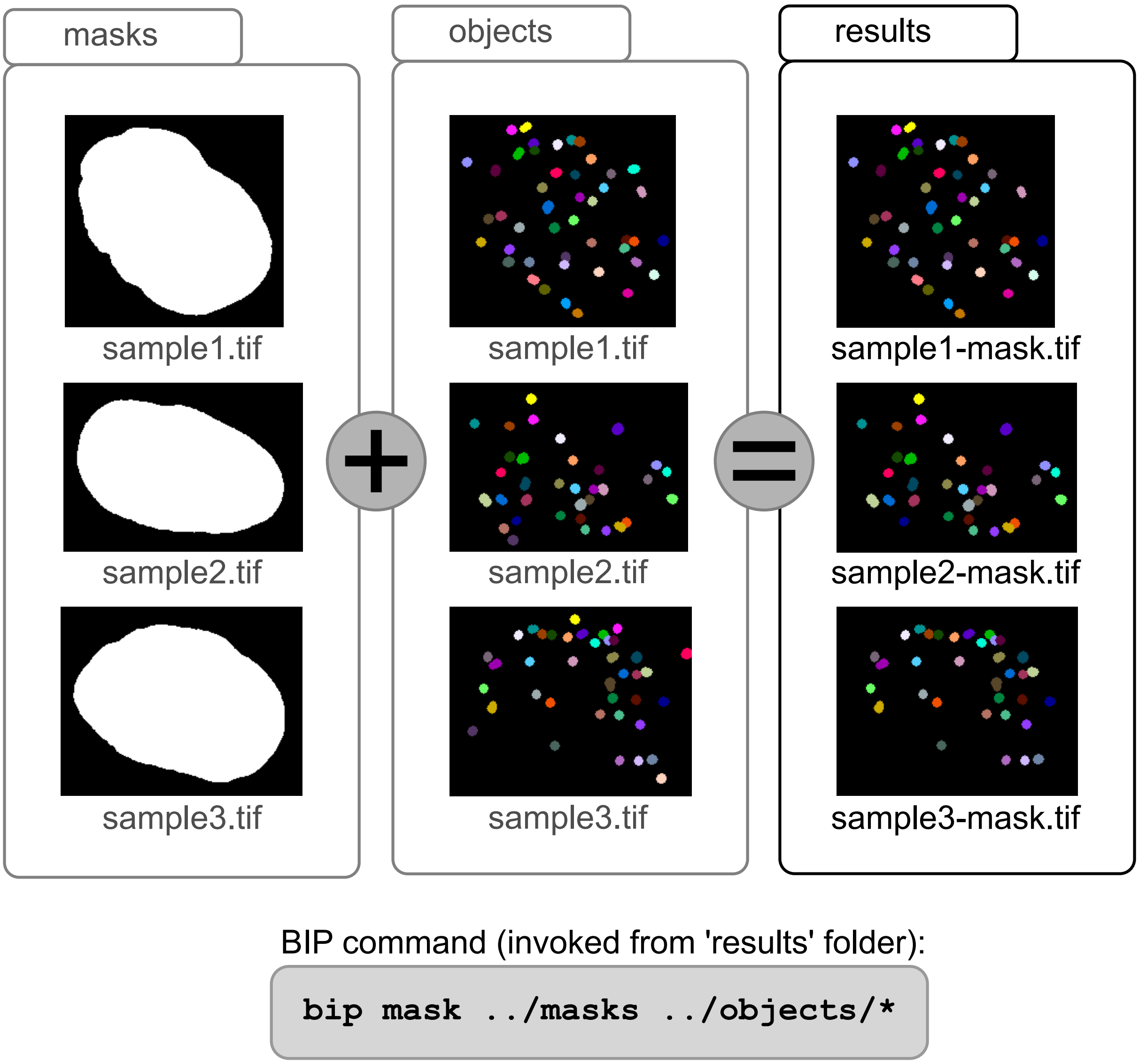

Figure S1: Using BIP for batch processing with combinations between different images having matched filenames. In this example, the objective is to remove objects (typically obtained by segmentation of a fluorescence channel) not contained within the corresponding masks (obtained by segmenting another channel). Paired object and mask image files have identical names. Using BIP, the operation is performed over a batch of images in a simple, single instruction.

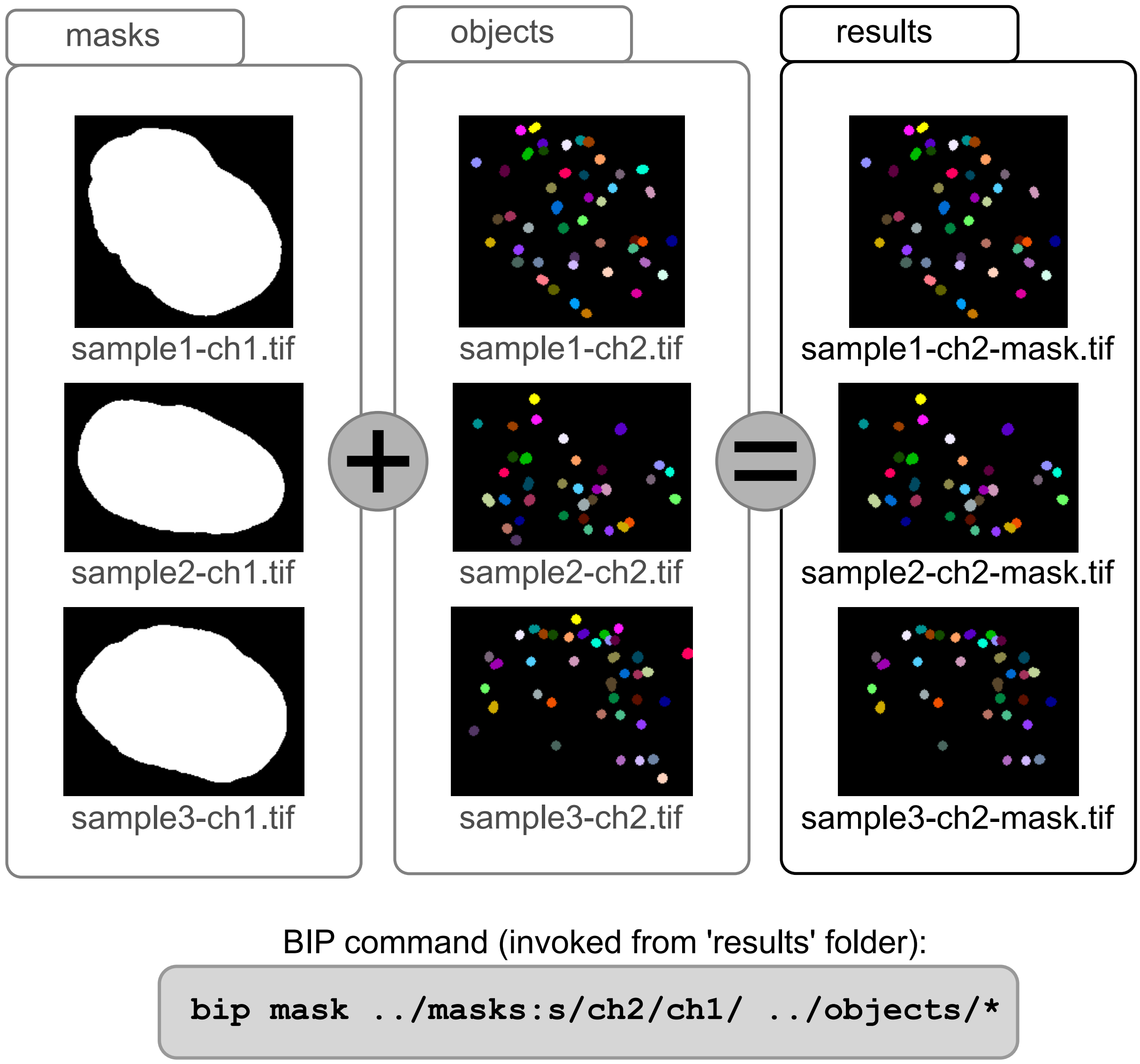

Figure S2: Using BIP for batch processing with combinations between different images having matched filenames, with substitutions. In this example, the objective is to remove objects (typically obtained by segmentation of a fluorescence channel) not contained within the corresponding masks (obtained by segmenting another channel). Paired object and mask image files differ by the channel number. Using BIP, the operation is performed over a batch of images in a simple, single instruction. A regular expression is used to generate the associated filename of each input filename (substituting 'ch02' by 'ch01'). If it was needed, several substitution patterns could be specified to map input filenames to their paired counterparts.

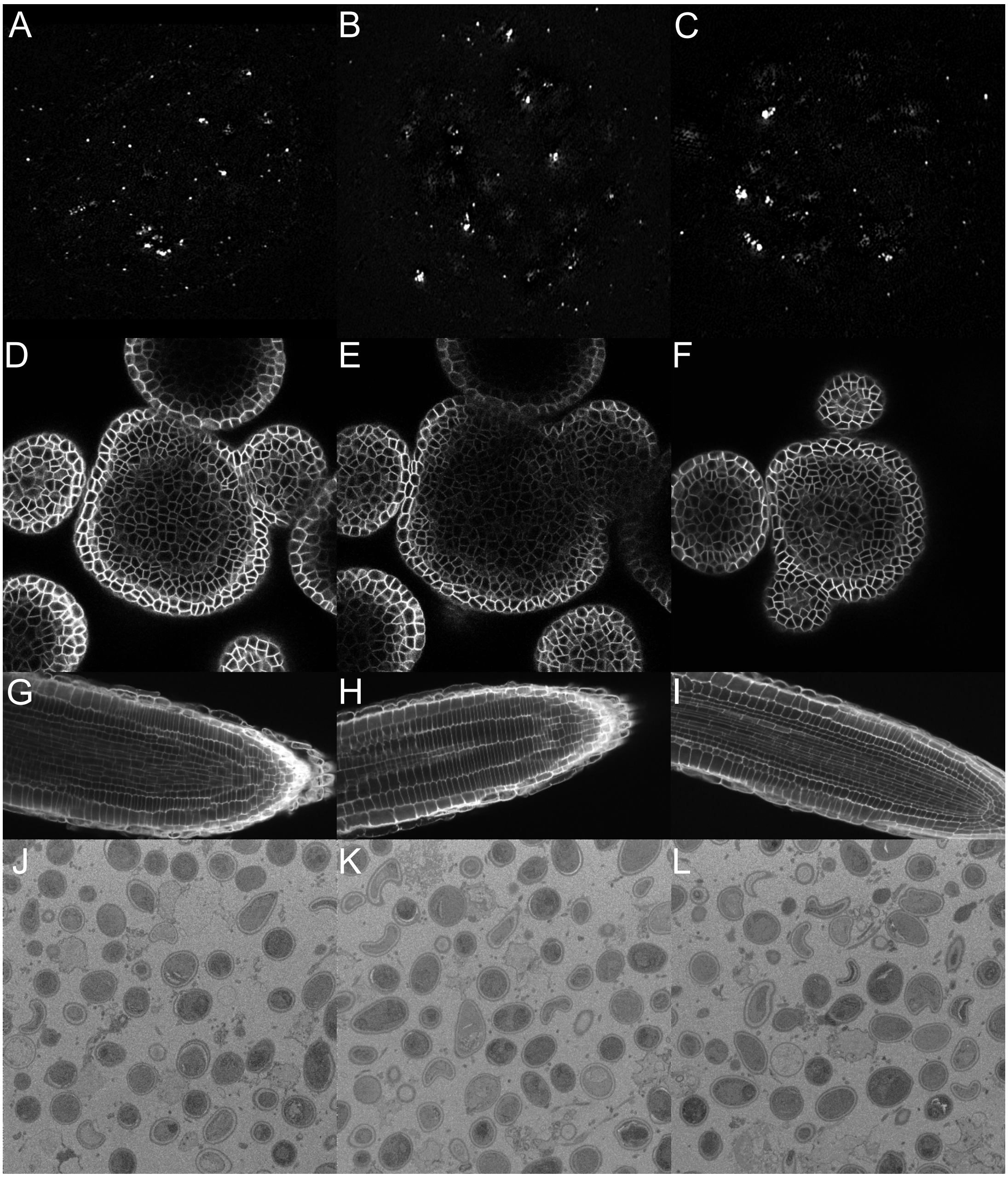

Figure S3: Image collection used for benchmarking: (ABC) 3D-SIM images of human telomeres; (DEF) 3D confocal images of shoot apical meristems; (GHI) 3D confocal images of root tips; (JKL) 2D SBF-SEM micrographs of *Anncaliia algerae* spores. See Table S3 for details and references. For 3D images, 2D sections are shown.

References

Chang, R., Davydov, A., Jaroenlak, P., Budaitis, B., Ekiert, D. C., Bhabha, G., and Prakash, M. (2023). Energetics of the microsporidian polar tube invasion machinery. *eLife*, **12**, RP86638.

Keller, D., Stinus, S., Umlauf, D., Gourbeyre, E., Biot, E., Olivier, N., Mahou, P., Beaurepaire, E., Andrey, P., and Crabbe, L. (2024). Non-random spatial organization of telomeres varies during the cell cycle and requires LAP2 and BAF. *iScience*, **27**, 109343.

Legland, D., Arganda-Carreras, I., and Andrey, P. (2016). MorphoLibJ: integrated library and plugins for mathematical morphology with ImageJ. *Bioinformatics*, **32**, 3532–3534.

McCormick, M. M., Liu, X., Ibanez, L., Jomier, J., and Marion, C. (2014). ITK: enabling reproducible research and open science. *Frontiers in Neuroinformatics*, **8**.

Schindelin, J., Arganda-Carreras, I., Frise, E., Kaynig, V., Longair, M., Pietzsch, T., Preibisch, S., Rueden, C., Saalfeld, S., Schmid, B., Tinevez, J.-Y., White, D. J., Hartenstein, V., Eliceiri, K., Tomancak, P., and Cardona, A. (2012). Fiji: an open-source platform for biological-image analysis. *Nature Methods*, **9**, 676–682.

van der Walt, S., Schönberger, J. L., Nunez-Iglesias, J., Boulogne, F., Warner, J. D., Yager, N., Gouillart, E., Yu, T., and the scikit-image contributors (2014). scikit-image: image processing in Python. *PeerJ*, **2**, e453.

Yoo, T. S., Ackerman, M. J., Lorensen, W. E., Schroeder, W., Chalana, V., Aylward, S., Metaxas, D., and Whitaker, R. (2002). Engineering and algorithm design for an image processing API: a technical report on ITK–the Insight Toolkit. *Studies in Health Technology and Informatics*, **85**, 586–592.
